# The role of gonadotrophin-releasing hormone antagonists in the treatment of patients with advanced hormone-dependent prostate cancer in the UK

**DOI:** 10.1007/s00345-016-1818-2

**Published:** 2016-04-20

**Authors:** Derek J. Rosario, Patrick Davey, James Green, Damien Greene, Bruce Turner, Heather Payne, Mike Kirby

**Affiliations:** 1 Royal Hallamshire Hospital, University of Sheffield, Sheffield, UK; 2 Northampton General Hospital, Northampton, UK; 3 Barts Health Hospitals, London, UK; 4 Sunderland Royal Hospital, Sunderland, UK; 5 Homerton University Hospital, London, UK; 6 University College Hospital, London, UK; 7 University of Hertfordshire, Hatfield, UK

**Keywords:** ADT, GnRH, Prostate cancer, Degarelix, UK, Cardiovascular

## Abstract

**Purpose:**

Comparing gonadotrophin-releasing hormone (GnRH) antagonists and agonists as androgen deprivation therapy for advanced prostate cancer (PC).

**Methods:**

This article stems from a round-table meeting in December 2014 to compare the properties of GnRH agonists and antagonists in the published literature in order to identify the patient groups most likely to benefit from GnRH antagonist therapy. A broad PubMed and congress abstract search was carried out in preparation for the meeting to ensure that the latest data and opinion were available for the discussions.

**Results:**

In randomised, controlled trials, GnRH antagonist therapy provides more rapid suppression of luteinising hormone, follicle-stimulating hormone and testosterone than GnRH agonist treatment. Compared with the GnRH agonist, there is evidence of improved disease control by a GnRH antagonist, with longer interval to prostate-specific antigen progression and greater reduction of serum alkaline phosphatase. In a post hoc analysis of six randomised trials, the risk of cardiac events within 1 year of initiating therapy was significantly lower among men receiving GnRH antagonist than agonist. Pre-clinical laboratory data suggest a number of mechanisms whereby GnRH antagonist therapy may benefit men with pre-existing cardiovascular disease (CVD), the most plausible hypothesis being that, unlike GnRH agonists, GnRH antagonists do not activate T lymphocytes, which act to increase atherosclerotic plaque rupture.

**Conclusion:**

When making treatment decisions, clinicians should consider comorbidities, particularly CVD, in addition to effects on PC. GnRH antagonists may be appropriate in patients with significant CV risk, existing osteopenia, lower urinary tract symptoms and significant metastatic disease.

## Introduction

Prostate cancer (PC) is the second most common cancer in men worldwide, with approximately 1.09 million men diagnosed in 2012, accounting for 15 % of all cancers in men [1]. In the UK, it is the most common cancer in men, with approximately 45,400 diagnoses and 10,600 deaths attributed to PC in 2012 [1]. Additionally, men with PC represent a high-risk population for cardiovascular disease, with many of the risk factors for PC being associated with high risk of CVD (e.g. obesity, diet, sedentary lifestyle). The commonest non-PC cause of death in such men is CVD.

Since the 1940s, the first-line treatment for advanced PC has been ADT, after it was demonstrated that surgical castration resulted in significant clinical improvement [2]. The irreversibility of the surgical procedure, along with its understandable lack of popularity with patients and clinicians, has led to increased use of chemical castration, with GnRH agonists being introduced in the 1980s, followed by GnRH antagonists in 2003 [3].

Recent epidemiological evidence has linked ADT with increased non-PC mortality seemingly due to increased CV mortality. Studies comparing orchiectomy with GnRH agonists suggest that agonist therapy may have morbidity above that seen with surgical castration. Most recently, a meta-analysis of a number of comparative trials between GnRH agonists and antagonists has revealed fundamental differences in outcomes between the two classes of drug. The purpose of the current paper is to provide an up to date review of the potential risks and benefits of the options available for ADT, so as to guide clinicians and patients as to the best likely option in any given situation.

## Rationale for use of ADT

The overall aim of ADT in advanced PC is to reduce testosterone levels, thereby minimising an important stimulus to androgen-sensitive PC cells and causing them to undergo apoptosis [3, 4]. ADT delays disease progression [5] and can also result in the dramatic reduction of skeletal metastases, decreased post-void residual urine and improved quality of life (QoL) [6].

## GnRH agonists

The GnRH agonists currently available share broadly similar overall survival outcomes [3] and achieve similar clinical improvements, such as reductions in bone pain, spinal cord compression risk and ureteral obstruction in the longer term [7]. Typically, GnRH agonists are indicated for the treatment of locally advanced/metastatic PC, as well as neo-adjuvant or adjuvant use with radiotherapy in high-risk localised or locally advanced PC [8].

**Fig. 1:**
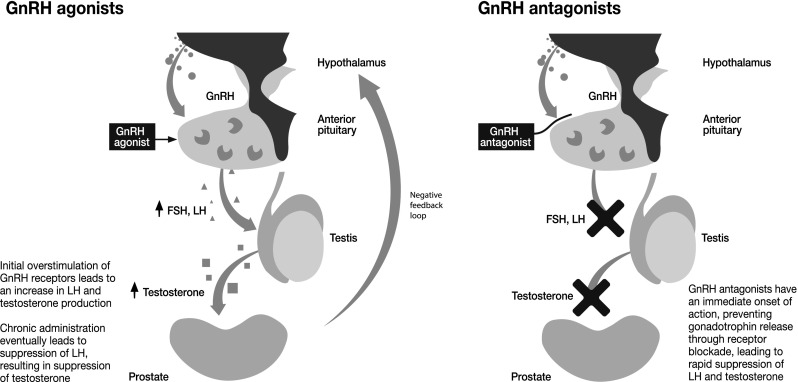
Contrasting modes of action of GnRH agonists and antagonists

Although GnRH agonists are the current standard of care, there are several factors relating to their mechanism of action to consider when selecting appropriate patients (Fig. 1). GnRH agonists work by overstimulating GnRH receptors, resulting in receptor desensitisation over time, with a consequent reduction in luteinising hormone (LH) and follicle-stimulating hormone (FSH) production, and reduced testosterone production [9] (Fig. 1). Testosterone suppression is only achieved after an initial LH surge that not only delays the testosterone reaching castrate levels, but also stimulates overproduction of testosterone for the first 30 days [9]. This potentially results in transient tumour expansion and a resultant flare in clinical symptoms, including worsened bone pain, urinary obstruction, spinal cord compression and potential CV effects [3, 9, 10]. Additionally, 5–17 % of men do not achieve acceptable testosterone suppression of ≤50 ng/dL [11], and 13–34 % do not achieve the more widely accepted modern threshold of 20 ng/dL [12–14].

## GnRH antagonists

A major advantage of the GnRH antagonists is their immediate onset of action, with more rapid and sustained suppression of testosterone than the GnRH agonists [15]. This is evidenced by rapid reduction in local symptoms [9] and in PSA. Furthermore, antagonists do not elicit the testosterone surge associated with agonists [16] obviating the need to add anti-androgens to the treatment regimen [15].

When degarelix (the only GnRH antagonist currently licensed in the UK) efficacy was compared with leuprorelin in a 12-month, randomised, open-label study, rapid testosterone suppression (testosterone levels ≤0.5 ng/mL by 3 days) was achieved in 96.1 % of patients, compared with none in the leuprolide group [15]. This rapid and predictably sustained testosterone suppression, which avoids the negative clinical effects associated with flare, more closely resembles the ‘gold standard’ ADT of surgical castration [16].

## The role of FSH and the need for sustained FSH suppression

The rapid testosterone suppression caused by degarelix was preceded by a similarly rapid decrease in LH and FSH levels that was maintained until the end of the study [15]. Furthermore, in the follow-up crossover study, FSH was further suppressed in patients switching from leuprolide to degarelix, with levels reaching those in patients receiving continuous degarelix treatment throughout the initial study [17].

FSH suppression is potentially important because of FSH’s role in tumour growth, bone resorption and regulation of adipocytes and obesity [18]. FSH-receptor-positive blood vessels have been identified in a 10-mm-thick tissue layer that extends into and outside the PC tumour mass [19]. Location of these cells in normal tissue immediately adjacent to tumour tissue is consistent with the hypothesis that tumour cells at the invasive front attract surrounding blood vessels to the tumour, with FSH-receptor expression being activated and consequently driving the proliferative process [19]. There may be additional benefits to suppressing FSH: the presence of FSH receptors in bone tissue has been implicated in accelerated bone resorption in post-menopausal women with raised FSH [20].

## PSA monitoring

Since its identification, PSA remains a controversial biomarker for PC screening [21]; however, the use of PSA as a marker for treatment response, prognosis and monitoring of disease progression is widely accepted [22–24]. The rate of PSA decline (PSA half-life) may also be of prognostic significance [25, 26]: **i**n a study of 153 patients receiving hormonal therapy, Lin et al. [25] found that shorter PSA half-life (≤0.5 months) was associated with significantly longer progression-free survival (PFS) and overall survival (OS) (median 24.6 and 48 months, respectively) than longer PSA half-life (median 17.2 and 43 months, respectively).

In the initial degarelix study and the subsequent crossover study, improved PSA-PFS associated with degarelix was also associated with delayed progression to castration-resistant PC (CRPC). PSA failure (defined as two consecutive PSA increases ≥50 % of nadir and ≥5 ng/mL in two consecutive measurements at least 2 weeks apart) [9] occurred mainly in patients with advanced disease and exclusively in patients with baseline PSA >20 ng/mL [22, 27]. In patients with baseline PSA >20 ng/mL, PSA recurrence was significantly less frequent for those receiving degarelix than for those receiving leuprolide (*p* = 0.04) [22]. Time to PSA failure or death in 25 % of these patients was also significantly longer for degarelix than for leuprolide (514 vs. 303 days; *p* = 0.01), i.e. progression or death was delayed by approximately 7 months longer with degarelix than with leuprolide [28].

The stronger correlation of PSA progression with survival in patients with hormone-sensitive PC receiving ADT, compared with patients with CRPC receiving chemotherapy is partly influenced by the disease’s natural history and the efficacy of available therapies in each setting [24]. Clinically, the goal of delaying PSA progression is important: PSA progression and subsequent emergence of castrate-resistant disease may trigger a move to chemotherapy, with its potential physical and psychological morbidities [16].

## Serum alkaline phosphatase

Bone metastases in PC are usually osteoblastic (i.e. they cause bone deposition), but these cancer cells can affect bones in two contrasting ways. Osteoclastic activity involves bone being broken down without new bone being formed, leading to osteolytic or lytic lesions that leave bone vulnerable to fracture. Osteoblastic activity involves new bone being formed without old bone being broken down first. The affected areas of the bones, osteoblastic or blastic lesions, become harder (sclerosis), but at the same time more brittle. This bone activity leads to elevated levels of parathyroid hormone (PTH), which promotes the growth and invasiveness of PC cells in bone. Thus, blastic metastases induce a vicious cycle in which PTH induces resorption of normal bone to support growth of bone metastases elsewhere [29].

In PC, elevated S-ALP levels appear to be predictive of progression of bone metastases and early mortality, with S-ALP at 6 months after treatment initiation giving better prediction of survival than baseline S-ALP [30]. An exploratory S-ALP analysis from the degarelix phase 3 study found that after initial peaks in all treatment groups, S-ALP was significantly more suppressed in patients with metastatic PC by degarelix than by leuprolide (96 vs. 179 IU/L; *p* = 0.014) [31]. Thus, degarelix may offer better S-ALP control than leuprolide and improve control of skeletal metastases over a 1-year period [31].

## Initiating ADT for advanced PC

ADT for advanced PC should always be tailored to the individual patient. Those experiencing symptoms such as spinal/bone pain or lower urinary tract symptoms (LUTS) should be assessed as to whether they might benefit from the more rapid reduction in testosterone, without associated testosterone and potential tumour flare, offered by a GnRH antagonist.

The clinical benefits of rapid testosterone reduction to castrate levels include reduction in intensity of lumbar back pain [32] and resolution of hydronephrosis and other LUTS [33]. Although the extension study from the degarelix phase 3 study has suggested some improvement in PSA-PFS [17], there are limited data available to make any firm conclusions regarding the benefits of switching between ADT modalities. Patients who experience an increase in PSA during ADT should have their testosterone levels retested before making further treatment decisions. Some patients may still be hormone responsive and therefore may be able to benefit from a switch to antagonist therapy [17].

## Adverse events associated with ADT

Chemical ADT is associated with a number of adverse events (AEs), including CVD, cognitive function effects [34–36], skeletal events, muscular pain, general pain and LUTS [7, 14]. In the CS21 phase 3 study of degarelix versus leuprolide, both agents were generally well tolerated, with a similar incidence of treatment-related AEs (79 and 78 % of patients in the degarelix and leuprolide groups, respectively) [15]. Most reported AEs were of mild-to-moderate intensity. Of note, degarelix was associated with a significantly higher incidence of injection site reactions (ISR) than leuprorelin (40 vs. <1 %; *p* = 0.001), with reactions occurring predominantly after the initiation dose [15]. This difference may have been due to the method of injection (subcutaneous for degarelix, intramuscular for leuprorelin), and the fact that reactions occurred most frequently with the initiation dose may be because a double injection is required for treatment initiation; an ISR was rarely seen with subsequent injections.

Additionally, chemical ADT is associated with sexual dysfunction [37], weight gain [15] and metabolic syndrome, and may be associated with raised triglycerides and cholesterol, reduced insulin sensitivity and type 2 diabetes [38, 39]. Weight gain, elevated triglycerides and type 2 diabetes are all CV risk factors. ADT has also been associated with an increased risk of CV events, compared with orchidectomy, including arterial embolic or thrombotic events, haemorrhagic or ischaemic cerebrovascular conditions, myocardial infarction (MI), heart failure and other ischaemic heart diseases [4, 40–44].

## Management of patients with increased CV risk

Given the risk factors for development of invasive PC and the patient demographics in PC, it is likely that at least one-third will have pre-existing vascular disease [40]. Furthermore, CVD is the most common cause of death in men with PC who do not die of the disease itself [45].

In 2010, the US Food and Drug Administration (FDA) asked manufacturers of GnRH analogues to add extra safety information to drug labels concerning the increased risk of diabetes and certain CV diseases (heart attack, sudden cardiac death, stroke) in men with PC [46], based on the epidemiological data [41]. Men with a history of CVD are most at risk, with agonists being significantly associated with increased risk of all-cause morbidity in men with a history of CVD-induced congestive heart failure or MI, but not in those with no comorbidities or only one CV risk factor [42].

In an analysis of pooled data from six phase 3, prospective, randomised trials of GnRH agonists versus antagonist involving more than 2300 men with PC [40], antagonist-based ADT appears to halve the number of cardiac events in men with pre-existing CVD during the first year of treatment, compared with agonists. In men with pre-existing CVD, there was a 56 % lower risk of a cardiac event (absolute risk reduction 8.2 %) in the first year of initiating ADT with a GnRH antagonist than with a GnRH agonist (HR 0.44; 95 % CI 0.26–0.74; *p* = 0.002; number needed to treat = 12) [40]. The magnitude of this risk reduction can be put into context by considering treatments intended to reduce risk in populations at high risk of CV events. For example, the landmark 4S simvastatin study demonstrated a 34 % relative risk reduction versus placebo over 5.4 years [47].

## Potential mechanisms of increased CV risk associated with GnRH agonists

GnRH agonists and antagonists both induce castrate levels of testosterone, which is associated with increased CV risk [40]. Traditionally, this risk has been attributed to metabolic changes similar to those seen in metabolic syndrome, which is defined as the presence of three of the following: central obesity, hyperglycaemia, hypertension or elevated serum triglycerides and low high-density lipoprotein (HDL) cholesterol [38]. However, the metabolic effects of lowered testosterone levels are more likely to promote CVD in the long term than during the first year of ADT, and the differing pattern of CV events associated with GnRH agonists and antagonists suggests that lowered testosterone might not be the sole cause of CVD in patients receiving ADT [40].

An alternative explanation for the association of CVD with GnRH agonists is the destabilisation of existing vascular lesions: rupture of atherosclerotic plaques appears to be the cause of most acute CV events [48, 49]. In contrast to stable plaques, unstable plaques have a large lipid core, containing thrombogenic macrophages covered by a thin fibrous cap. Paradoxically, these vulnerable plaques often have a well-preserved lumen. T lymphocytes may destabilise vulnerable fibrous caps in two ways. Firstly, releasing pro-inflammatory cytokines prevents synthesis of collagen required to maintain the fibrous cap. Secondly, releasing cytokine CD40L stimulates infiltrating macrophages to secrete collagenases that degrade the fibrous cap [48]. These T lymphocytes express GnRH receptors that are sensitive to GnRH agonists and antagonists [50, 51]. GnRH agonist is likely to lead to increased proliferation and activity of T cells [52], with resulting fibrotic cap disruption and plaque instability. By contrast, a GnRH antagonist would not cause increased proliferation or activity, and plaque stability would be maintained.

## CV risk stratification

CV risk can inform treatment decisions for ADT and guide CV risk reduction strategies, such as lifestyle modification (e.g. smoking cessation, diet and exercise), antihypertensive therapy, lipid-lowering therapy (e.g. statins) and antiplatelet therapy. GPs are generally well versed in this area, and the additional CV risk among some patients receiving GnRH agonists should be highlighted to GPs so they can provide appropriate management.

**Fig. 2:**
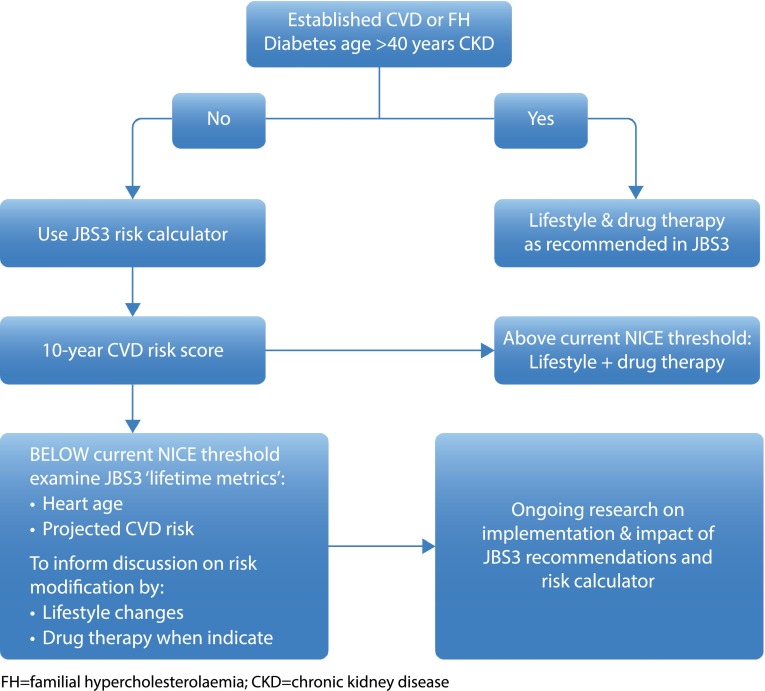
JBS3 algorithm for CV risk calculator

The new Joint British Societies (JBS3) risk calculator tool [53] is being promoted for use in conjunction with a healthcare provider, but may also be used by patients alone. The key message of the tool is that reducing risk factors by making lifestyle changes will provide long-term benefits and extend the patient’s healthy life. Conversely, the JBS3 risk calculator may prevent a patient who otherwise has optimal risk factors being recommended a statin on the basis of age alone (Table 1; Fig. 2).

**Table 1:** Key recommendations from the JBS3 risk calculator tool

The risk calculator is not appropriate for diabetic patients aged over 40 years, those with CKD Stages 3–5 or those with FH
A non-fasting blood sample should be used for lipid profile estimation, measuring total cholesterol and HDL cholesterol
Non-HDL cholesterol should be used in preference to LDL cholesterol as the treatment goal for lipid-lowering therapy
Non-HDL cholesterol is based on non-fasting total cholesterol minus HDL cholesterol
Patients with existing CVD require intensive risk factor modification with diet, lifestyle and drug therapy, without the need for estimation of future risk This also applies to those with diabetes older than 40 years and those with CKD Stages 3–5 or FH
Clear lifestyle guidance is provided on a healthy diet and physical activity, with support for an increase in exercise on referral and community-based exercise initiatives
All healthcare professionals should be able to Ask and Assess adiposity and Advise (The 3 As) appropriate adult patients on evidence-based ways to lose weight
The lipid recommendations endorse the use of non-HDL cholesterol and all high-risk patients should receive professional lifestyle support to reduce total cholesterol, raise HDL cholesterol and lower triglycerides

*CKD* chronic kidney disease, *FH* familial hypercholesterolaemia, *HDL* high-density lipoprotein, *LDL* low-density lipoprotein

## Management of CV and other risk factors

Having assessed CV risk, clinicians should consider ADT options accordingly:

- In the low-CV-risk patient, clinicians should select the best therapy for the individual based on disease characteristics
- In the presence of pre-existing or significant CV risk, consider the use of degarelix

Once an increased CV risk has been identified, patients should not only be offered the optimal ADT modality for their risk status, but should also be offered support in managing their CV condition. Options include pharmacological therapy to reduce CV risk (antihypertensives, statins, etc.) and professional support with lifestyle interventions, including diet, activity/exercise and smoking cessation advice [53]. ADT modality should be considered particularly carefully in light of recent research demonstrating the potential survival benefits associated with use of chemotherapy alongside ADT (for hormone-sensitive metastatic disease). One consequence of improved survival in this setting is longer-term exposure to ADT and potentially to increased CV risk [54, 55].

Given that PC patients tend to be aged 65 years or older and that agonist-based ADT is associated with osteopenia and increased fracture risk [3], it may be appropriate to offer bone densitometry testing using dual energy X-ray absorptiometry (DEXA). Blood tests may also be appropriate to diagnose diabetes mellitus and metabolic syndrome.

Overall, attention should be paid to the patient’s QoL and personal treatment goals. As cancer treatments improve and patients are living for longer following diagnosis, there has been an increasing awareness of the need to provide the care and support required for them to lead healthy and active lives. The NICE prostate cancer guidelines CG175 [8] state that all men receiving ADT should be prescribed a 12-week supervised exercise intervention consisting of aerobic and resistance exercise. Whereas this recommendation is based primarily on demonstration of improved QoL and reduction in fatigue [56], mechanistic evidence exists suggesting improvement in endothelial function from such an intervention, thus a possible impact on reducing CV risk [57].

## Conclusions

PC remains the most frequently occurring male cancer in the UK. The majority of cases of PC respond, at least initially, to suppression of testosterone by the available options for ADT. Although GnRH agonists have become established the most common option, they have certain disadvantages, not least being the initial surge in testosterone, which may have serious clinical implications. The more recently available GnRH antagonists provide similar testosterone suppression, but have the major advantage of more rapid suppression of testosterone, brought about by blockage of the GnRH signal to the pituitary, with consequent rapid decreases in PSA, LH and FSH levels. The shortened PSA half-life, compared with agonists, may be of prognostic significance [9]. Antagonist-based testosterone suppression is also more predictably sustained in the longer term, with less breakthrough than seen with agonists, and may offer improved disease control, compared with GnRH agonists [15].

Antagonists are also associated with a reduced risk of CV events in men with pre-existing CVD, compared with agonists. Mechanisms by which the different CVD risks of agonists and antagonists may occur are still being elucidated, but it seems increasingly likely that GnRH agonists stimulate T cell-mediated pro-inflammatory responses, leading to destabilisation of atherosclerotic plaques.

Treatment decisions in PC should always be based on a number of considerations, including an individual patient’s disease characteristics, other comorbidities and his treatment preferences. Given the demographics of the patient population, it is reasonable to assume that a degree of CVD may be present, although it may be sub-clinical. By carrying out a simple series of tests, it is possible to assess an individual patient’s CVD risk. We are entering a time of more individualised care, with a greater tailoring of treatment to each patient. Where we have choice, such as in the ADT phase of PC treatment, habit no longer has a place. All options should be considered, discussed and selected based on the benefits and risks associated with each. For patients with advanced PC, starving the tumour of testosterone is key, but the method by which this is achieved should also take into account the individual patient’s disease characteristics and other circumstances. In those men with underlying conditions, such as CVD or osteopenia, choice of ADT approach should be guided by the additional risk associated with those conditions.
